# 1-(2-Bromo­benz­yl)-3-isopropyl­benz­imid­azolin-2-one

**DOI:** 10.1107/S1600536809042871

**Published:** 2009-10-23

**Authors:** Sudesh T. Manjare, Ray J. Butcher, Nidhi Goel, Udai P. Singh, Harkesh B. Singh

**Affiliations:** a Department of Chemistry, Indian Institute of Technology Bombay, Powai, Mumbai 400 076, India; b Department of Chemistry, Howard University, 525 College Street NW, Washington DC 20059, USA; c Department of Chemistry, Indian Institute of Technology Roorkee, Roorkee 247 667, India

## Abstract

In the structure of the title compound, C_17_H_17_BrN_2_O, the central phenyl and imidazol-2-one rings are coplanar (dihedral angle between planes of 0.73 (11)°). The angles subtended by the substituents on the N atoms of the imidazol-2-one ring range from 109.71 (14)° to 128.53 (15) due to steric hindrance of these substituents with the phenyl H atoms. The carbonyl O and Br both make two weak C—H⋯O and C—H⋯Br inter­actions with two adjacent mol­ecules, thus forming an three-dimensional array.

## Related literature

For benzimidazolones as precursors to important pharmacologically active compounds, see: Biagi *et al.* (2001 ▶). For the benzimidazolones as sources of stable carbenes, see: Albéniz *et al.* (2002 ▶); Denk *et al.* (2001 ▶); Jarrar & Fataftah (1977 ▶); Manjare *et al.* (2009 ▶); Çetinkaya *et al.* (1998 ▶). For the preparation, see: Kuhn *et al.* (1996 ▶). 
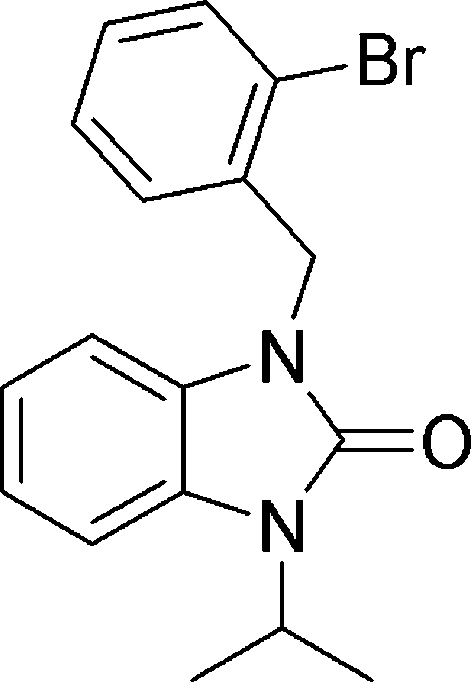


## Experimental

### 

#### Crystal data

C_17_H_17_BrN_2_O
*M* *_r_* = 345.24
Monoclinic, 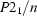

*a* = 12.1417 (3) Å
*b* = 10.1146 (3) Å
*c* = 12.2008 (3) Å
β = 95.763 (1)°
*V* = 1490.79 (7) Å^3^
*Z* = 4
Mo *K*α radiation
μ = 2.76 mm^−1^
*T* = 203 K
0.38 × 0.24 × 0.17 mm

#### Data collection

Bruker APEXII diffractometer
Absorption correction: multi-scan (*SADABS*; Sheldrick, 1996 ▶) *T*_min_ = 0.631, *T*_max_ = 0.746
26524 measured reflections
3771 independent reflections
3039 reflections with *I* > 2σ(*I*)
*R*_int_ = 0.072

#### Refinement

*R*[*F* ^2^ > 2σ(*F* ^2^)] = 0.029
*wR*(*F* ^2^) = 0.075
*S* = 0.99
3771 reflections
192 parameters
H-atom parameters constrained
Δρ_max_ = 0.75 e Å^−3^
Δρ_min_ = −0.42 e Å^−3^

### 

Data collection: *APEX2* (Bruker, 2005 ▶); cell refinement: *SAINT* (Bruker, 2005 ▶); data reduction: *SAINT*; program(s) used to solve structure: *SHELXS97* (Sheldrick, 2008 ▶); program(s) used to refine structure: *SHELXL97* (Sheldrick, 2008 ▶); molecular graphics: *SHELXTL* (Sheldrick, 2008 ▶); software used to prepare material for publication: *SHELXL97*.

## Supplementary Material

Crystal structure: contains datablocks I, global. DOI: 10.1107/S1600536809042871/om2287sup1.cif

Structure factors: contains datablocks I. DOI: 10.1107/S1600536809042871/om2287Isup2.hkl

Additional supplementary materials:  crystallographic information; 3D view; checkCIF report

## Figures and Tables

**Table 1:** Hydrogen-bond geometry (Å, °)

*D*—H⋯*A*	*D*—H	H⋯*A*	*D*⋯*A*	*D*—H⋯*A*
C3—H3⋯O1^i^	0.94	2.52	3.439 (2)	167
C9—H9⋯O1^ii^	0.94	2.50	3.374 (2)	154
C10—H10⋯Br1^iii^	0.94	3.05	3.6457 (18)	123
C15—H15⋯Br1^iv^	0.99	3.11	3.7941 (18)	128

Symmetry codes: (i) 
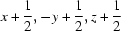
; (ii) 
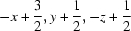
; (iii) 
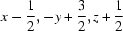
; (iv) 
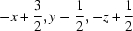
.

**Fig. 1.**
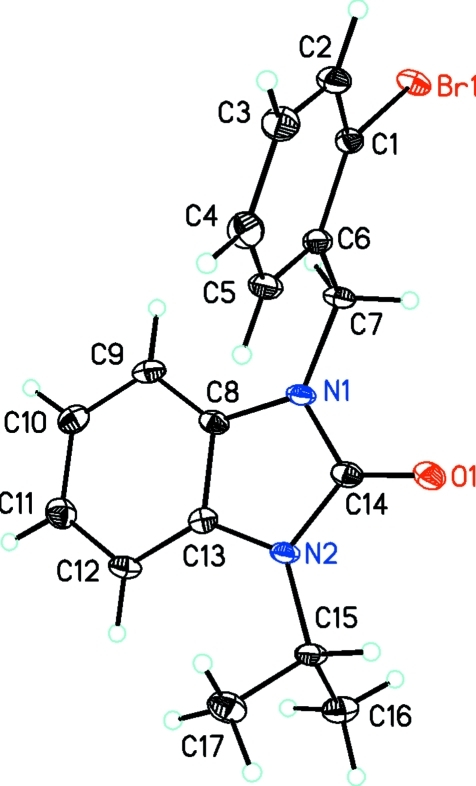
The molecular structure of C17H17BrN2O the showing the atom numbering scheme and 50% probability displacement ellipsoids.

**Fig. 2.**
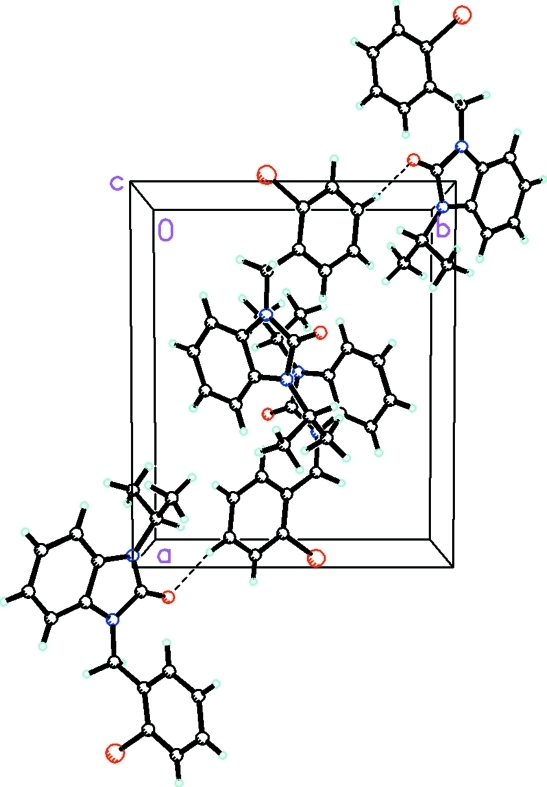
The molecular packing for C17H17BrN2O viewed down the c axis. The C—H···O interactions are shown by dashed lines.

## supplementary crystallographic information

### Comment

There has been much interest in benzimidazolones as precursors to important pharmacologically active compounds (Biagi *et al.* 2001) and also as precursors to stable carbene derivatives (Albéniz *et al.*2002; Çetinkaya *et al.* 1998; Denk *et al.* 2001; Manjare *et al.* 2009). We report the structure of the title compound, C_17_H_17_BrN_2_O, 1, prepared as part of a study of the reactivity of related selenones (Manjare *et al.* 2009) and with a view to stabilizing a monomeric selenium dioxide derivative. The product was obtained as the minor product from the selenone by reaction with H_2_O_2_ (Fig. 3).

As shown in Figure 1, the central phenyl and imidazol-2-one rings are coplanar (dihedral angle between planes of 0.73 (11)°). The benzoimidazol-2-one moiety is thus a planar system (r.m.s. deviation from plane of 0.0069 (1) Å). The phenyl ring of the substituent bromobenzyl group makes a dihedral angle of 80.64 (3) with this plane. In addition the C atoms attached to N1 and N2 are also coplanar with this ring. Within the imidazole ring the C—N and C—C distances range from 1.380 (2) to 1.402 (2) and thus are significantly shorter than single bonds. However, the bonds from N1 and N2 to the C atoms of the substituents are 1.468 (2) and 1.448 (2) which are in the range found for C—N single bonds. Thus the bond lengths in this ring are similar to those found in structures of dihydro-imidazol-2-one derivatives (Denk *et al.*, 2001). The angles subtended by the substituents on the N's of the imidazol-2-one ring range from 109.71 (14)° to 128.53 (15) due to steric hindrance of these substituents with the phenyl H atoms.

The carbonyl O and Br both make two weak C–H···O and C–H···Br interactions with two adjacent molecules thus forming an 3-D array.

### Experimental

The title compound was obtained by the addition of hydrogen peroxide (0.21 ml, 1.82 mmol) to the solution of selenone 2 (0.15 g, 0.37 mmol; Kuhn *et al.*, 1996) in chloroform (15 ml) at room temperature. Sodium sulfate was added to the reaction mixture then the solution was filtered and evaporated. Compound 1 was obtained as minor product along with the compound 3 (Scheme 1).

### Refinement

H atoms were placed in geometrically idealized positions and constrained to ride on their parent atoms with C—H distances of 0.95 and 0.99 Å and U_iso_(H) = 1.2U_eq_(C) [1.5 U_eq_(CH_3_).

### Crystal data

**Table:**

C_17_H_17_BrN_2_O	*F*(000) = 704
*M**_r_* = 345.24	*D*_x_ = 1.538 Mg m^−^^3^
Monoclinic, *P*2_1_/*n*	Mo *K*α radiation, λ = 0.71073 Å
Hall symbol: -P 2yn	Cell parameters from 9903 reflections
*a* = 12.1417 (3) Å	θ = 2.6–28.4°
*b* = 10.1146 (3) Å	µ = 2.76 mm^−^^1^
*c* = 12.2008 (3) Å	*T* = 203 K
β = 95.763 (1)°	Prism, colorless
*V* = 1490.79 (7) Å^3^	0.38 × 0.24 × 0.17 mm
*Z* = 4	

### Data collection

**Table:**

Bruker APEXII diffractometer	3771 independent reflections
Radiation source: fine-focus sealed tube	3039 reflections with *I* > 2σ(*I*)
graphite	*R*_int_ = 0.072
ω scans	θ_max_ = 28.6°, θ_min_ = 3.0°
Absorption correction: multi-scan (*SADABS*; Sheldrick, 1996)	*h* = −14→16
*T*_min_ = 0.631, *T*_max_ = 0.746	*k* = −12→13
26524 measured reflections	*l* = −16→16

### Refinement

**Table:**

Refinement on *F*^2^	Primary atom site location: structure-invariant direct methods
Least-squares matrix: full	Secondary atom site location: difference Fourier map
*R*[*F*^2^ > 2σ(*F*^2^)] = 0.029	Hydrogen site location: inferred from neighbouring sites
*wR*(*F*^2^) = 0.075	H-atom parameters constrained
*S* = 0.99	*w* = 1/[σ^2^(*F*_o_^2^) + (0.0471*P*)^2^] where *P* = (*F*_o_^2^ + 2*F*_c_^2^)/3
3771 reflections	(Δ/σ)_max_ = 0.003
192 parameters	Δρ_max_ = 0.75 e Å^−^^3^
0 restraints	Δρ_min_ = −0.42 e Å^−^^3^

### Special details

**Table:**

Geometry. All e.s.d.'s (except the e.s.d. in the dihedral angle between two l.s. planes) are estimated using the full covariance matrix. The cell e.s.d.'s are taken into account individually in the estimation of e.s.d.'s in distances, angles and torsion angles; correlations between e.s.d.'s in cell parameters are only used when they are defined by crystal symmetry. An approximate (isotropic) treatment of cell e.s.d.'s is used for estimating e.s.d.'s involving l.s. planes.
Refinement. Refinement of *F*^2^ against ALL reflections. The weighted *R*-factor *wR* and goodness of fit *S* are based on *F*^2^, conventional *R*-factors *R* are based on *F*, with *F* set to zero for negative *F*^2^. The threshold expression of *F*^2^ > σ(*F*^2^) is used only for calculating *R*-factors(gt) *etc*. and is not relevant to the choice of reflections for refinement. *R*-factors based on *F*^2^ are statistically about twice as large as those based on *F*, and *R*- factors based on ALL data will be even larger.

### Fractional atomic coordinates and isotropic or equivalent isotropic displacement parameters (Å^2^)

**Table:**

	*x*	*y*	*z*	*U*_iso_*/*U*_eq_	
Br1	1.037577 (14)	0.582633 (17)	0.249055 (14)	0.01832 (8)	
O1	0.61458 (11)	0.41194 (12)	0.14806 (11)	0.0195 (3)	
N1	0.66453 (12)	0.58527 (14)	0.26835 (12)	0.0145 (3)	
N2	0.48981 (12)	0.52025 (15)	0.25058 (12)	0.0141 (3)	
C1	0.95844 (14)	0.46626 (18)	0.33498 (14)	0.0139 (4)	
C2	1.01703 (15)	0.37113 (19)	0.39732 (14)	0.0178 (4)	
H2	1.0942	0.3642	0.3966	0.021*	
C3	0.96085 (16)	0.28571 (18)	0.46116 (15)	0.0199 (4)	
H3	0.9996	0.2203	0.5041	0.024*	
C4	0.84746 (16)	0.29766 (19)	0.46107 (15)	0.0200 (4)	
H4	0.8092	0.2402	0.5045	0.024*	
C5	0.78948 (15)	0.39346 (17)	0.39770 (15)	0.0172 (4)	
H5	0.7124	0.4002	0.3989	0.021*	
C6	0.84372 (14)	0.47977 (17)	0.33241 (14)	0.0140 (4)	
C7	0.78243 (15)	0.58255 (17)	0.25879 (15)	0.0159 (4)	
H7A	0.8135	0.6700	0.2777	0.019*	
H7B	0.7945	0.5642	0.1820	0.019*	
C8	0.60923 (14)	0.66479 (17)	0.33738 (13)	0.0137 (4)	
C9	0.64598 (14)	0.76802 (17)	0.40571 (14)	0.0164 (4)	
H9	0.7205	0.7948	0.4126	0.020*	
C10	0.56824 (15)	0.83047 (18)	0.46372 (15)	0.0186 (4)	
H10	0.5905	0.9015	0.5105	0.022*	
C11	0.45850 (16)	0.79053 (18)	0.45424 (15)	0.0191 (4)	
H11	0.4083	0.8339	0.4958	0.023*	
C12	0.42093 (15)	0.68751 (17)	0.38446 (14)	0.0167 (4)	
H12	0.3462	0.6617	0.3771	0.020*	
C13	0.49817 (14)	0.62453 (18)	0.32639 (13)	0.0137 (4)	
C14	0.59197 (14)	0.49597 (17)	0.21446 (14)	0.0147 (4)	
C15	0.39093 (14)	0.44538 (17)	0.20836 (15)	0.0154 (4)	
H15	0.4166	0.3691	0.1668	0.019*	
C16	0.31692 (15)	0.52811 (19)	0.12750 (16)	0.0207 (4)	
H16A	0.3569	0.5517	0.0654	0.031*	
H16B	0.2951	0.6079	0.1638	0.031*	
H16C	0.2515	0.4776	0.1017	0.031*	
C17	0.32998 (17)	0.38940 (19)	0.30151 (16)	0.0214 (4)	
H17A	0.3826	0.3466	0.3553	0.032*	
H17B	0.2755	0.3254	0.2718	0.032*	
H17C	0.2932	0.4606	0.3367	0.032*	

### Atomic displacement parameters (Å^2^)

**Table:**

	*U*^11^	*U*^22^	*U*^33^	*U*^12^	*U*^13^	*U*^23^
Br1	0.01045 (11)	0.02172 (12)	0.02339 (12)	−0.00227 (7)	0.00473 (7)	0.00112 (7)
O1	0.0122 (7)	0.0221 (7)	0.0245 (7)	0.0023 (5)	0.0027 (5)	−0.0052 (5)
N1	0.0074 (7)	0.0186 (8)	0.0172 (7)	−0.0002 (6)	0.0004 (6)	0.0008 (6)
N2	0.0073 (7)	0.0155 (8)	0.0195 (8)	0.0003 (6)	0.0009 (6)	−0.0027 (6)
C1	0.0107 (9)	0.0150 (9)	0.0160 (8)	−0.0022 (7)	0.0015 (7)	−0.0036 (7)
C2	0.0112 (9)	0.0207 (10)	0.0212 (9)	0.0027 (8)	0.0002 (7)	−0.0020 (8)
C3	0.0187 (10)	0.0192 (10)	0.0212 (9)	0.0063 (8)	−0.0010 (8)	0.0013 (7)
C4	0.0209 (10)	0.0196 (10)	0.0195 (9)	−0.0021 (8)	0.0027 (7)	0.0027 (7)
C5	0.0102 (9)	0.0216 (10)	0.0197 (9)	−0.0006 (7)	0.0014 (7)	0.0010 (7)
C6	0.0104 (9)	0.0144 (9)	0.0170 (9)	−0.0003 (7)	0.0001 (7)	−0.0029 (7)
C7	0.0080 (9)	0.0197 (10)	0.0200 (9)	−0.0001 (7)	0.0010 (7)	0.0023 (7)
C8	0.0097 (8)	0.0154 (9)	0.0159 (8)	0.0029 (7)	0.0007 (6)	0.0050 (7)
C9	0.0125 (9)	0.0166 (9)	0.0193 (9)	−0.0035 (7)	−0.0028 (7)	0.0037 (7)
C10	0.0189 (10)	0.0164 (9)	0.0197 (9)	−0.0019 (8)	−0.0016 (7)	−0.0020 (7)
C11	0.0179 (10)	0.0181 (10)	0.0215 (9)	0.0044 (8)	0.0030 (7)	−0.0006 (7)
C12	0.0091 (8)	0.0183 (9)	0.0226 (9)	0.0003 (7)	0.0011 (7)	0.0003 (7)
C13	0.0133 (9)	0.0121 (9)	0.0152 (8)	−0.0003 (7)	−0.0003 (7)	0.0015 (7)
C14	0.0109 (9)	0.0154 (9)	0.0176 (9)	0.0024 (7)	0.0001 (7)	0.0029 (7)
C15	0.0089 (9)	0.0151 (9)	0.0222 (9)	−0.0018 (7)	0.0010 (7)	−0.0025 (7)
C16	0.0138 (9)	0.0215 (10)	0.0258 (10)	−0.0002 (8)	−0.0024 (8)	−0.0001 (8)
C17	0.0178 (10)	0.0178 (10)	0.0293 (10)	−0.0032 (8)	0.0056 (8)	0.0015 (8)

### Geometric parameters (Å, °)

**Table:**

Br1—C1	1.9000 (18)	C7—H7B	0.9800
O1—C14	1.224 (2)	C8—C9	1.382 (2)
N1—C14	1.381 (2)	C8—C13	1.402 (2)
N1—C8	1.386 (2)	C9—C10	1.387 (3)
N1—C7	1.448 (2)	C9—H9	0.9400
N2—C14	1.380 (2)	C10—C11	1.386 (3)
N2—C13	1.400 (2)	C10—H10	0.9400
N2—C15	1.468 (2)	C11—C12	1.393 (3)
C1—C2	1.379 (3)	C11—H11	0.9400
C1—C6	1.397 (2)	C12—C13	1.386 (2)
C2—C3	1.388 (3)	C12—H12	0.9400
C2—H2	0.9400	C15—C16	1.518 (3)
C3—C4	1.382 (3)	C15—C17	1.526 (2)
C3—H3	0.9400	C15—H15	0.9900
C4—C5	1.387 (3)	C16—H16A	0.9700
C4—H4	0.9400	C16—H16B	0.9700
C5—C6	1.391 (2)	C16—H16C	0.9700
C5—H5	0.9400	C17—H17A	0.9700
C6—C7	1.519 (2)	C17—H17B	0.9700
C7—H7A	0.9800	C17—H17C	0.9700
			
C14—N1—C8	110.12 (14)	C10—C9—H9	121.4
C14—N1—C7	122.53 (15)	C11—C10—C9	121.48 (17)
C8—N1—C7	127.09 (15)	C11—C10—H10	119.3
C14—N2—C13	109.71 (14)	C9—C10—H10	119.3
C14—N2—C15	121.73 (14)	C10—C11—C12	121.47 (17)
C13—N2—C15	128.53 (15)	C10—C11—H11	119.3
C2—C1—C6	122.59 (16)	C12—C11—H11	119.3
C2—C1—Br1	118.37 (13)	C13—C12—C11	117.35 (16)
C6—C1—Br1	119.04 (14)	C13—C12—H12	121.3
C1—C2—C3	119.25 (17)	C11—C12—H12	121.3
C1—C2—H2	120.4	C12—C13—N2	132.55 (16)
C3—C2—H2	120.4	C12—C13—C8	120.75 (16)
C4—C3—C2	119.41 (17)	N2—C13—C8	106.70 (15)
C4—C3—H3	120.3	O1—C14—N2	127.12 (17)
C2—C3—H3	120.3	O1—C14—N1	126.46 (16)
C3—C4—C5	120.77 (17)	N2—C14—N1	106.41 (15)
C3—C4—H4	119.6	N2—C15—C16	110.71 (14)
C5—C4—H4	119.6	N2—C15—C17	111.76 (15)
C4—C5—C6	120.97 (17)	C16—C15—C17	112.89 (15)
C4—C5—H5	119.5	N2—C15—H15	107.0
C6—C5—H5	119.5	C16—C15—H15	107.0
C5—C6—C1	117.00 (16)	C17—C15—H15	107.0
C5—C6—C7	122.41 (16)	C15—C16—H16A	109.5
C1—C6—C7	120.56 (16)	C15—C16—H16B	109.5
N1—C7—C6	113.26 (14)	H16A—C16—H16B	109.5
N1—C7—H7A	108.9	C15—C16—H16C	109.5
C6—C7—H7A	108.9	H16A—C16—H16C	109.5
N1—C7—H7B	108.9	H16B—C16—H16C	109.5
C6—C7—H7B	108.9	C15—C17—H17A	109.5
H7A—C7—H7B	107.7	C15—C17—H17B	109.5
C9—C8—N1	131.17 (16)	H17A—C17—H17B	109.5
C9—C8—C13	121.76 (16)	C15—C17—H17C	109.5
N1—C8—C13	107.05 (15)	H17A—C17—H17C	109.5
C8—C9—C10	117.18 (16)	H17B—C17—H17C	109.5
C8—C9—H9	121.4		
			
C6—C1—C2—C3	−0.6 (3)	C10—C11—C12—C13	1.3 (3)
Br1—C1—C2—C3	179.61 (13)	C11—C12—C13—N2	−179.69 (18)
C1—C2—C3—C4	0.0 (3)	C11—C12—C13—C8	−0.6 (3)
C2—C3—C4—C5	0.3 (3)	C14—N2—C13—C12	178.84 (18)
C3—C4—C5—C6	0.2 (3)	C15—N2—C13—C12	0.7 (3)
C4—C5—C6—C1	−0.8 (3)	C14—N2—C13—C8	−0.3 (2)
C4—C5—C6—C7	177.72 (17)	C15—N2—C13—C8	−178.43 (16)
C2—C1—C6—C5	1.0 (3)	C9—C8—C13—C12	−0.1 (3)
Br1—C1—C6—C5	−179.22 (13)	N1—C8—C13—C12	−178.99 (15)
C2—C1—C6—C7	−177.50 (16)	C9—C8—C13—N2	179.17 (15)
Br1—C1—C6—C7	2.3 (2)	N1—C8—C13—N2	0.29 (18)
C14—N1—C7—C6	−80.5 (2)	C13—N2—C14—O1	−179.94 (17)
C8—N1—C7—C6	93.0 (2)	C15—N2—C14—O1	−1.7 (3)
C5—C6—C7—N1	3.0 (2)	C13—N2—C14—N1	0.22 (19)
C1—C6—C7—N1	−178.55 (15)	C15—N2—C14—N1	178.48 (14)
C14—N1—C8—C9	−178.90 (17)	C8—N1—C14—O1	−179.87 (17)
C7—N1—C8—C9	6.9 (3)	C7—N1—C14—O1	−5.4 (3)
C14—N1—C8—C13	−0.17 (19)	C8—N1—C14—N2	−0.03 (19)
C7—N1—C8—C13	−174.36 (15)	C7—N1—C14—N2	174.48 (15)
N1—C8—C9—C10	178.79 (17)	C14—N2—C15—C16	−104.36 (18)
C13—C8—C9—C10	0.2 (3)	C13—N2—C15—C16	73.5 (2)
C8—C9—C10—C11	0.4 (3)	C14—N2—C15—C17	128.87 (17)
C9—C10—C11—C12	−1.2 (3)	C13—N2—C15—C17	−53.2 (2)

### Hydrogen-bond geometry (Å, °)

**Table:**

*D*—H···*A*	*D*—H	H···*A*	*D*···*A*	*D*—H···*A*
C3—H3···O1^i^	0.94	2.52	3.439 (2)	167
C9—H9···O1^ii^	0.94	2.50	3.374 (2)	154
C10—H10···Br1^iii^	0.94	3.05	3.6457 (18)	123
C15—H15···Br1^iv^	0.99	3.11	3.7941 (18)	128

Symmetry codes: (i) *x*+1/2, −*y*+1/2, *z*+1/2; (ii) −*x*+3/2, *y*+1/2, −*z*+1/2; (iii) *x*−1/2, −*y*+3/2, *z*+1/2; (iv) −*x*+3/2, *y*−1/2, −*z*+1/2.

## References

[bb1] Albéniz, A. C., Espinet, P., Manrique, R. & Pérez-Mateo, A. (2002). *Angew. Chem. Int. Ed.* **41**, 2363–2366. 10.1002/1521-3773(20020703)41:13<2363::AID-ANIE2363>3.0.CO;2-9 12203593

[bb2] Biagi, G., Calderone, V., Giorgi, I., Livi, O., Scartoni, V., Baragatti, B. & Martinotti, E. (2001). *Farmaco*, **56**, 841–849. 10.1016/s0014-827x(01)01148-x 11765036

[bb3] Bruker (2005). *APEX2 *and**SAINT** Bruker AXS Inc., Madison, Wisconsin, USA.

[bb4] Çetinkaya, B., Çetinkaya, E., Chamizo, J. A., Hitchcock, P. B., Jasaim, H. A., Küçükbay, H. & Lappert, M. F. (1998). *J. Chem. Soc. Perkin Trans. 1*, pp. 2047–2054.

[bb5] Denk, M. K., Rodezno, J. M., Gupta, S. & Lough, L. J. (2001). *J. Organomet. Chem.* **617**, 242–253.

[bb6] Jarrar, A. A. & Fataftah, Z. (1977). *Tetrahedron*, **33**, 2127–2129.

[bb7] Kuhn, N., Fawzi, R., Kratz, T., Steimann, M. & Henkel, G. (1996). *Phosphorus Sulfur Silicon*, **112**, 107–119.

[bb8] Manjare, S. T., Singh, H. B. & Butcher, R. J. (2009). *Acta Cryst.* E**65**, o2640. 10.1107/S1600536809039130 PMC2971053 21578255

[bb9] Sheldrick, G. M. (1996). *SADABS* University of Göttingen, Germany.

[bb10] Sheldrick, G. M. (2008). *Acta Cryst.* A**64**, 112–122. 10.1107/S0108767307043930 18156677

